# Community mobilization to modify harmful gender norms and reduce HIV risk: results from a community cluster randomized trial in South Africa

**DOI:** 10.1002/jia2.25134

**Published:** 2018-07-04

**Authors:** Audrey Pettifor, Sheri A Lippman, Ann Gottert, Chirayath M Suchindran, Amanda Selin, Dean Peacock, Suzanne Maman, Dumisani Rebombo, Rhian Twine, Francesc Xavier Gómez‐Olivé, Stephen Tollman, Kathleen Kahn, Catherine MacPhail

**Affiliations:** ^1^ Department of Epidemiology University of North Carolina Gillings School of Global Public Health Chapel Hill NC USA; ^2^ Carolina Population Center University of North Carolina at Chapel Hill Chapel Hill NC USA; ^3^ MRC/Wits Rural Public Health and Health Transitions Research Unit (Agincourt) School of Public Health University of the Witwatersrand Johannesburg South Africa; ^4^ Center for AIDS Prevention Studies (CAPS) Department of Medicine University of California at San Francisco San Francisco CA USA; ^5^ Population Council HIV and AIDS program Washington DC USA; ^6^ Department of Biostatistics University of North Carolina Gillings School of Global Public Health Chapel Hill NC USA; ^7^ Sonke Gender Justice Cape Town South Africa; ^8^ School of Public Health Division of Social and Behavioural Science University of Cape Town Cape Town South Africa; ^9^ Department of Health Behavior University of North Carolina Gillings School of Global Public Health Chapel Hill NC; ^10^ Epidemiology and Global Health Unit Department of Public Health and Clinical Medicine Umeå University Umeå Sweden; ^11^ School of Health University of New England Armidale NSW Australia; ^12^ Wits RHI University of the Witwatersrand Johannesburg South Africa

**Keywords:** HIV, community mobilization, gender norms, South Africa, gender‐based violence

## Abstract

**Introduction:**

Community mobilization (CM) is increasingly recognized as critical to generating changes in social norms and behaviours needed to achieve reductions in HIV. We conducted a CM intervention to modify negative gender norms, particularly among men, in order to reduce associated HIV risk.

**Methods:**

Twenty two villages in the Agincourt Health and Socio‐Demographic Surveillance Site in rural Mpumalanga, South Africa were randomized to either a theory‐based, gender transformative, CM intervention or no intervention. Two cross‐sectional, population‐based surveys were conducted in 2012 (pre‐intervention, n = 600 women; n = 581 men) and 2014 (post‐intervention, n = 600 women; n = 575 men) among adults ages 18 to 35 years. We used an intent‐to‐treat (ITT) approach using survey regression cluster‐adjusted standard errors to determine the intervention effect by trial arm on gender norms, measured using the Gender Equitable Mens Scale (GEMS), and secondary behavioural outcomes.

**Results:**

Among men, there was a significant 2.7 point increase (Beta Coefficient 95% CI: 0.62, 4.78, *p* = 0.01) in GEMS between those in intervention compared to control communities. We did not observe a significant difference in GEMS scores for women by trial arm. Among men and women in intervention communities, we did not observe significant differences in perpetration of intimate partner violence (IPV), condom use at last sex or hazardous drinking compared to control communities. The number of sex partners in the past 12 months (AOR 0.29, 95% CI 0.11 to 0.77) were significantly lower in women in intervention communities compared to control communities and IPV victimization was lower among women in intervention communities, but the reduction was not statistically significant (AOR 0.53, 95% CI 0.24 to 1.16).

**Conclusion:**

Community mobilization can reduce negative gender norms among men and has the potential to create environments that are more supportive of preventing IPV and reducing HIV risk behaviour. Nevertheless, we did not observe that changes in attitudes towards gender norms resulted in desired changes in risk behaviours suggesting that more time may be necessary to change behaviour or that the intervention may need to address behaviours more directly.

**Clinical Trials number:**

ClinicalTrials.gov NCT02129530.

## 1 Introduction

Young people in sub‐Saharan Africa (SSA) face an incredibly high risk of HIV infection. In many parts of SSA young women made up 26% of all new HIV infections in 2016 despite comprising only 10% of the population 1. Men also have a high prevalence in the region, are less likely to engage with prevention and care services than women, and have largely been overlooked in the HIV prevention response 2, 3, 4, 5. There is increasing consensus that social and cultural norms which condone gender inequity underlie many of the key factors driving HIV risk for both women and men, including multiple and age‐disparate sexual partnerships, alcohol abuse and gender‐based violence 6, 7. In South Africa, it has been well documented that normative masculine narratives condone multiple partnerships and physical and sexual violence within sexual partnerships 8, 9, 10, 11. Such social contexts result in young women experiencing high rates of gender‐based violence further contributing to the high levels of HIV infection observed 12, 13, 14, 15.

Community mobilization (CM) strategies, or programmes that encourage community dialogue and action around shared concerns 16, are increasingly being recognized as a necessary element or “critical enabler” needed to activate changes indispensable to achieve large‐scale reductions in HIV 17. Interventions that have used CM strategies have demonstrated success in changing social norms, including modifying harmful gender norms, and fostering social environments that are more supportive of safer sexual behaviours 14, 18, 19, 20, 21, 22, 23, 24, 25, 26, 27. However, community mobilization as a concept has been largely under‐defined in the HIV prevention arena and has lacked theoretical frameworks that could help guide the development, implementation and replication/scale‐up of effective mobilization programmes. To date, very few CM programmes for gender transformation and HIV prevention have been designed around a defined conceptual model of community mobilization or been rigorously evaluated. 16, 28, 29

To address this gap, we conducted a cluster randomized trial of a novel, theory‐based gender transformative CM intervention to change gender norms that place young women and men at risk of HIV acquisition; it is among the first trials to use a defined mobilization model 16. The primary objectives of the CM intervention were to increase awareness about the relationship between gender inequities and HIV and encourage the community, especially men, to take action both in their own lives and in their communities to address negative gender norms and associated HIV risk.

## 2 Methods

### 2.1 Trial design

We conducted a cluster randomized trial to measure the impact of the intervention on the study outcomes. Two cross‐sectional, population‐based surveys with 1181 (n = 600 women and 581 men) and 1175 (n = 600 women and 575 men) adults ages 18 to 35 years were completed to measure the effect of the intervention on study outcomes and CM domains in each community. The cross‐sectional surveys were conducted first at baseline, prior to intervention implementation (March to June 2012) and then after two years of intervention (July to November 2014) 30. A serial cross‐sectional evaluation design was chosen as we hypothesized the CM intervention would impact change in held beliefs or norms at the community level and activities targeted the full community, whereas a panel design would measure impact on changing norms within a defined cohort. Serial cross sections have advantages for measuring community‐level indicators and community change 31, 32, 33.

### 2.2 Community randomization

Communities were randomized at a 1:1 ratio, resulting in 11 intervention communities and 11 control communities. In order to achieve a balanced allocation of control and intervention communities with respect to covariates hypothesized to be associated with the primary outcome, views towards gender norms, a restricted randomization scheme was employed 34. This process is described in the published protocol 30.

### 2.3 Study setting

The study setting for this community RCT includes 22 villages in the Agincourt Health and socio‐Demographic Surveillance System (Agincourt HDSS) site in the rural Bushbuckridge sub‐District in Mpumalanga province of South Africa 35. HIV prevalence in Mpumalanga is estimated to be 21.8% among adults ages 15 to 49 36 and prevalence in the study area was over 45% among 35 to 39 year olds in 2010 to 2011 37.

### 2.4 Baseline and post‐intervention assessments

The sampling strategy was designed to ensure equal numbers of male and female participants. Using the Agincourt HDSS annual census in the year prior to each survey for the sampling frame, we first created two strata of households with 18‐ to 35‐year‐old male and female residents; we then randomly sampled from the two strata (male and female) in order to reach a target enrolment for each community of 55 individuals with 27/28 males and females per community. Eligibility criteria for survey participation included: being a resident (spends a majority of nights in a 7‐day week within the home), being 18‐ to 35‐year old as per confirmed date of birth, and being a permanent resident in the study area for the past 12 months prior to the sampling. The surveys, offered in either English or Shangaan (the local language), were interviewer‐administered using Computer Assisted Self‐Interview (CAPI). No compensation was provided for participation in the survey.

### 2.5 Intervention

The CM intervention was developed in partnership with the South African non‐governmental organization Sonke Gender Justice, based on their One Man Can Campaign. Details of the intervention development are described in the trial protocol 30 and intervention materials are available online (http://www.genderjustice.org.za). One Man Can was created in 2006 as “a programme to promote healthy, equitable relationships and support men and boys to take action to end domestic and sexual violence” 30. The intervention was delivered through local community mobilizers and through volunteer cadres called Community Action Teams (CATs), who were trained to support activities in each community. Intervention activities comprised workshops, community activities and leadership engagement open to men and women, with workshops and activity content focused on seven areas; (1) gender, power, and health, (2) gender and violence, (3) alcohol abuse, (4) gender, HIV and AIDS, (5) healthy relationships, (6) human rights, and (7) taking action for change. Workshops were intensive 2‐day activities and each workshop (there were 5 workshop agendas) included activities that addressed each of the 7 content areas. Mobilizers and CAT members created opportunities for community dialogue about these themes within and outside of workshops through community activities focusing on developing a shared concern and critical consciousness around HIV and gender. Examples of community activities included door‐to‐door home visits, street soccer and soccer tournaments, mural design and discussions, facilitated discussions in venues where men gather including shabeens/bars, and film screenings with a thematic discussion to follow, and community theatre. The team also engaged formal leadership and community organizations in dialogue on the intervention content and sought support for intervention activities. Intervention components served as a catalyst to mobilize community members to take action in their own lives and to become change agents in their communities around gender equity, gender‐based violence and HIV prevention.

**Figure 1:**
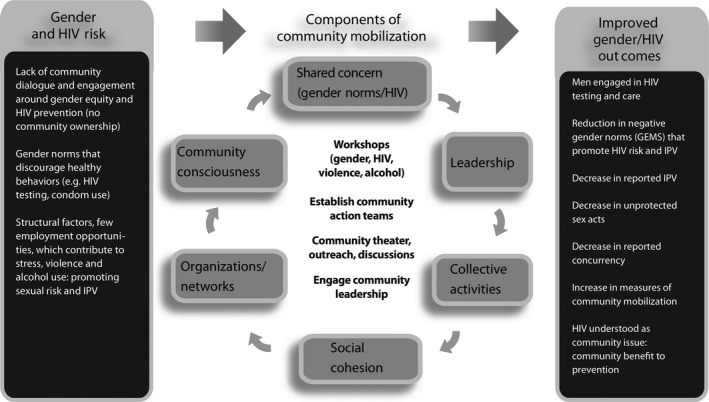
Conceptual Framework for Community Mobilization Intervention.

Intervention components also mapped onto a theoretical framework for CM comprising six target domains (Figure 1). The domains were identified through review and synthesis of key features needed to successfully mobilize communities drawn from mobilization‐related literature from the following disciplines: social movements (sociology), community empowerment, community development and community capacity 16.

The resulting domains, hypothesized to create change in a community included: (1) development of a shared concern (around HIV and gender norms); (2) building critical consciousness; (3) establishing and leveraging organizations and groups, including links to networks; (4) engaging leadership (individual and/or institutional); (5) engaging communities in collective activities/actions; and (6) building social cohesion. 16, 28, 30

The Intervention was implemented by a team of two supervisors, community mobilizers and trained CAT members, in each intervention community. Mobilizers underwent an intensive initial month‐long training including intervention content and facilitation skills as well as ongoing trainings as needs were identified.

The target population for the intervention was young adults ages 18 to 35 (the resident target population was approximately 25,000 individuals), with a focus on men, though all adult community members were welcomed. Implementation targets were set to ensure that a minimum number of activities were delivered in each community and detailed activity tracking data were collected to monitor targets, intervention reach and activity mix. Targets were set to reach 20% of 18‐ to 35‐year‐old male intervention community residents with at least one workshop in the first year and 40% by the end of the second year. Reaching 40% of the population by the end of the intervention was chosen as an appropriate target as it is nearly three times the 15% commonly thought of as a “critical mass” of a population's opinion leaders for successful diffusion of health behaviour change messages 38.

### 2.6 Measures

Intervention randomization assignment at the community level was the primary exposure used for all primary analysis. In a secondary analysis, we also sought to explore whether the intervention was effective among those who reported exposure *versus* those who may have resided in the intervention communities but had not interacted with the intervention itself. For this ancillary analysis we utilized a measure of reported intervention exposure compiled by items assessed in the endline survey. We coded self‐reported participation with each possible One Man Can activity as binary (0,1), with the exception of the number of workshops attended, which had four categories. We fit a 1‐parameter partial credit model to participants in intervention villages to assess item fit 39, and then generated weighted maximum likelihood estimate (WLE) scores for all participants (control and intervention) from this model 40. Individual intervention exposure was then categorized into low, medium and high based on observed cut‐points or natural exposure groups, that represent no active participation in the intervention (low), participation in limited or more passive activities, like video presentations (medium), and engagement in more active One Man Can activities, including participating in rallies or multiple workshops, or being a CAT member (high).

The primary outcome for assessing the intervention impact was views towards gender norms, as measured by the Gender Equitable Men's Scale (GEMS) 41, 42. Since its development in 2008, GEMS has been used in dozens of studies in sub‐Saharan Africa, and has consistently achieved reliability and investigators have reported associations of GEMS with hypothesized HIV risk behaviours among both men and women—including in our study area 11, 26, 43, 44, 45, 46, 47, 48. Adaptation and validation of the scale are described elsewhere for men 49, and a similar process was followed up for women. We based the scale on a 24‐item Ethiopian adaptation of GEMS that had achieved high internal consistency reliability and associations with outcomes of interest among men and women 46. We adapted some item phrasing in consultation with the local research team to increase appropriateness for the local social context. The set of final items for men and women was selected through exploratory and confirmatory factor analyses using the 2012 data set. Confirmatory factor analyses demonstrated adequate model fit and good internal consistency reliability, with an alpha of 0.79 for men and 0.76 for women at baseline, and 0.76 for men and 0.71 for women at endline. Items fell into four content areas relevant to the intervention: sexual relationships (e.g. “Men are always ready to have sex”); violence (e.g. “A woman should tolerate violence to keep her family together”); reproductive health and disease prevention (e.g. “It is a woman's responsibility to avoid getting pregnant”); and household roles and decision‐making (e.g. “A man should have the final word about decisions in his home”). Individuals’ scores were the sum of all items (each item ranged from 1 to 3: “agree a lot”(1), “somewhat agree”(2), and “do not agree at all”(3)). Men's scores ranged from 17 to 51 (17 items); women's scores ranged from 13 to 39 (13 items). Higher scores represent more equitable views towards gender norms; therefore individuals with a high GEMS score (e.g., over 40 for men) tended to respond “do not agree at all” to items such as those noted above.

Secondary endpoints for the trial included having multiple (two or more) sexual partners in the last 12 months, condom use at last sex, perpetration or enacting of intimate partner violence (IPV) and victimization or experience of IPV in the last 12 months, and recent hazardous/harmful drinking assessed using the Alcohol Use Disorder Identification Test (AUDIT) 50. IPV victimization and perpetration were defined as reporting at least one of seven types of physical or sexual IPV on a World Health Organization questionnaire adapted for South Africa 12, 51. Hazardous/harmful drinking was defined as an AUDIT score of 8 or above.

### 2.7 Ethics and informed consent

This study was approved by the Institutional Review Boards at the University of North Carolina‐Chapel Hill and University of California‐San Francisco, the Human Research Ethics Committee (Medical) at the University of the Witwatersrand in South Africa, and the Mpumalanga Department of Health and Social Development Research Committee. Written informed consent either in English or Shangaan was obtained from all participants. The authors declare that they have no competing interests.

### 2.8 Sample size

Power and sample size were calculated to detect changes in the primary endpoint of interest, the GEMS score, in men and women separately. A total sample size of 1200 (600 participants per arm) would result in 91% power in men and 80% power in women to detect a four‐point increase (men) and three‐point increase (women) in GEMS between arms, assuming an intra‐cluster correlation of 0.05 and an average cluster size of 55 resulted in an estimated design effect of 3.7.

### 2.9 Statistical analysis

Descriptive and inferential analyses were performed on the data using SAS version 9.3 (SAS Institute Inc., Cary, NC, USA). All analyses were conducted separately for men and women and were weighted to account for sampling probability and to represent the distribution of men and women aged 18 to 35 years in Agincourt based on the 2011 Agincourt HDSS census. An intent to treat (ITT) approach was used to determine the effect of the intervention on the primary and secondary outcomes, with individual as the unit of analysis. Clustering of the main outcome (GEMS score) by village was lower than anticipated at baseline (Intraclass Correlation (ICC) = 0.03; Design Effect (DEFF) = 2.4) and endline (ICC < 0.01; DEFF = 1.3; after adjusting for intervention status).

For the main analysis we assessed changes in gender norms by randomization arm using survey regression with robust variance estimators to account for clustering and ensure correct standard errors. The specified model included the intervention status (intervention or control), the round (baseline or endline), and the interaction between intervention and round. The effect estimate comes from the interaction term, which reflects the difference in changes over time by study arm. We adjusted the models for demographic variables hypothesized to be associated with outcomes: age, education, marital status and receipt of income in last three months. As a sensitivity analysis to assess the robustness of our findings to model selection, we employed a Generalized Linear Mixed Model (GLMM) for the primary outcome for men and women—results were very similar (see Appendix S1).

To assess differences in the primary and secondary outcomes by individuals’ level of dose/exposure (low, medium, high) to the intervention, ancillary analyses were conducted on the sample of individuals in intervention communities at endline. Estimates for each outcome (mean/%) were generated for each level of exposure to the intervention, and a Wald chi‐square test was conducted to assess differences between any of the three groups.

### 2.10 Role of the funding source

The funders of this study played no role in the design, analysis or interpretation of the results of the study. The corresponding author had full access to all the data in the study and had final responsibility for the decision to submit for publication.

## 3 Results

The CM intervention was successfully rolled out in the 11 intervention communities in July 2012 and was concluded in July 2014. Summary implementation data are included in the Appendix S1. By the end of Year 2 an average of 37.3% of 18‐ to 35‐year‐old men in each intervention community were reached with at least one 2‐day workshop and an estimated 5830 activities were conducted in intervention villages. Notably, contamination was low: only 4% of control residents in the final survey reported any One Man Can engagement, as compared to 54% of intervention village residents who reported engagement.

**Figure 2:**
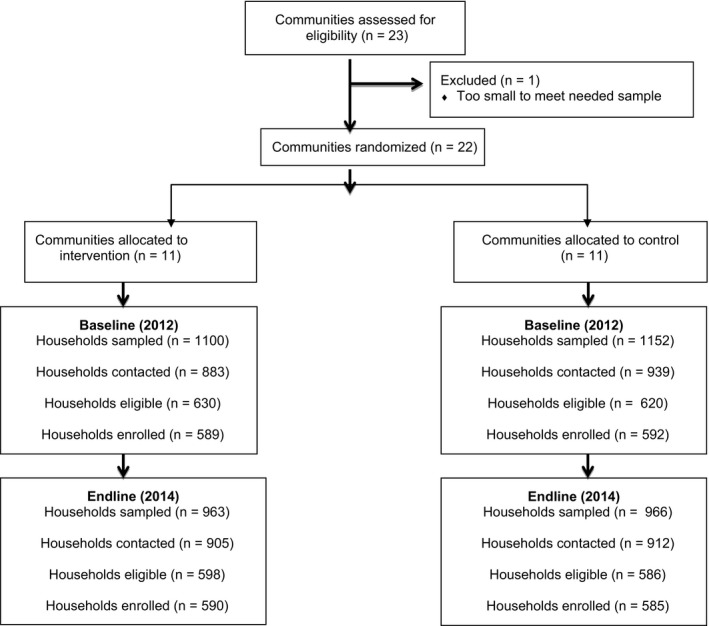
Trial profile.

In the baseline survey in 2012, a total of 2252 households were sampled. Contact was made with 1822 households (81%). Among the households contacted, 69% had an eligible resident (n = 1250); almost all ineligibility was due to non‐residence in the study site in the past 12 months. Among those eligible, 1181 people (n = 600 women and 581 men) were enrolled into the study (94%) and 69 (6%) refused to participate (Figure 2).

In the endline survey in 2014, a total of 1929 households were sampled. Contact was made with 1817 households (94%). Among the households contacted, 65% had an eligible resident (n = 1184); again almost all ineligibility was due to non‐residence in the study site. Among those eligible, 1175 people (n = 600 women and 575 men) were enrolled into the study (99%), 9 (1%) refused to participate (Figure

Intervention and control communities were similar with respect to key socio‐demographic characteristics at baseline; there were no statistically significant differences (Table 1). Across arms, the mean age of participants was 22 years of age, about a quarter to a third had completed high school, most had not earned any income in the last 3 months and the vast majority of men were unmarried (87.6% to 92.8%) while about a quarter of women reported being married. There was a statistically significant difference for GEMS scores among men (*p* < 0.01) at baseline, with control communities reporting higher GEMS scores than intervention communities. No other outcomes, including GEMS scores for women or secondary outcomes, differed by study arm at baseline.

**Table 1:** Baseline and endline demographic characteristics stratified by gender and study arm May to July 2012 and May to July 2014, Agincourt Health and Socio‐Demographic Surveillance Site, South Africa

Demographic characteristics	Baseline		Endline	
	Control n = 592	Intervention n = 589	Control n = 585	Intervention n = 590
	Weighted % (95% CI)	Weighted % (95% CI)	Weighted % (95% CI)	Weighted % (95% CI)
Age (mean)				
Men	22.2 (21.6, 22.7)	22.6 (21.4, 23.8)	23.6 (23.0, 24.2)	23.8 (22.8, 24.7)
Women	25.2 (24.4, 26.0)	25.2 (24.9, 25.5)	26.1 (25.2, 27.0)	25.5 (24.8, 26.2)
Completed high school				
Men	25.6 (19.0, 32.3)	31.8 (22.7, 40.9)	35.6 (29.8, 41.5)	32.0 (22.9, 41.2)
Women	34.2 (26.3, 42.2)	42.3 (28.8, 55.8)	34.9 (28.1, 41.8)	41.9 (33.9, 49.8)
Married (*vs*. other)				
Men	7.2 (4.3, 10.0)	12.4 (6.2, 18.7)	12.6 (6.1, 19.1)	11.0 (5.0, 17.0)
Women	33.0 (29.5, 36.6)	26.4 (17.4, 35.5)	29.1 (19.7, 38.4)	32.3 (27.7, 36.9)
Received any income in past three months				
Men	28.7 (20.8, 36.5)	34.7 (25.6, 43.7)	31.9 (18.7, 45.1)	23.4 (19.4, 27.5)
Women	35.9 (28.8, 43.0)	41.6 (34.1, 49.0)	32.7 (26.0, 39.4)	34.6 (27.5, 41.7)

In the primary analysis, we observed a significant difference in GEMS scores over time between intervention and control communities among men. Among men, there was a 2.7 point increase (Beta Coefficient 95% CI: 0.62, 4.78) in GEMS scores between survey participants in intervention as compared to control communities over time (Table 2). We did not observe a significant difference in GEMS scores for women in intervention compared to control communities (Table 3). Results of the sensitivity analysis using GLMM were substantively equivalent to findings using cluster‐adjusted survey regression (Appendix S1).

**Table 2:** Among men, primary and secondary outcomes at baseline and endline and adjusted effect estimates, stratified by study arm

	Baseline		Endline		Adjusted effect estimate^a^	
	Control n = 289	Intervention n = 292	Control n = 287	Intervention n = 288		
Characteristics	Weighted mean (95% CI)	Weighted mean (95% CI)	Weighted mean (95% CI)	Weighted mean (95% CI)	Beta (95% CI)	*p* value
Gender equitable men's scale (GEMS)^b^						
Possible range: 17 to 51	36.5 (35.5, 37.5)	34.4 (33.4, 35.4)	34.5 (33.3, 35.4)	35.2 (34.4, 36.0)	2.70 (0.62, 4.78)	0.01
	**Weighted % (95% CI)**	**Weighted % (95% CI)**	**Weighted % (95% CI)**	**Weighted % (95% CI)**	**AOR (95% CI)**	***p*** **value**
Sexual behaviour						
Multiple partners in past 12 months	35.1 (27.2, 44.0)	41.2 (33.6, 49.2)	30.9 (24.7, 37.8)	29.8 (20.7, 40.8)	0.73 (0.32, 1.67)	0.46
Condom use at last sex^c^	42.6 (34.1, 51.5)	38.5 (33.8, 43.4)	46.5 (41.3, 51.8)	49.8 (39.5, 60.1)	1.35 (0.88, 2.07)	0.17
Intimate partner violence (IPV)						
Victim of IPV in past 12 months	5.1 (3.1, 8.3)	6.3 (4.0, 9.7)	4.9 (1.3, 16.2)	4.8 (3.3, 6.9)	0.80 (0.18, 3.49)	0.77
Perpetrated IPV in past 12 months	9.6 (6.8, 13.4)	12.3 (7.6, 19.3)	8.2 (5.2, 12.6)	10.4 (7.0, 15.1)	0.99 (0.52, 1.89)	0.97
Substance use						
Hazardous, harmful drinking	23.4 (16.5, 32.2)	24.5 (18.6, 31.7)	12.6 (7.5, 20.4)	19.0 (11.8, 29.1)	1.53 (0.43, 5.39)	0.51

All estimates controlled for age, education, marital status and received any income in the last three months. AOR, adjusted odds ratio.

a Effect estimates represent the difference between intervention and control at endline, net of the difference at baseline.

b Higher scores represent more equitable views towards gender norms. n = 1155 (1 missing).

c Among those reporting ever having had sex (vaginal or anal), n = 1005.

**Table 3:** Among women, primary and secondary outcomes at baseline and endline and adjusted effect estimates, stratified by study arm

	Baseline		Endline		Adjusted effect estimate^a^	
	Control n = 303	Intervention n = 297	Control n = 298	Intervention n = 302		
Characteristics	Weighted mean (95% CI)	Weighted mean (95% CI)	Weighted mean (95% CI)	Weighted mean (95% CI)	Beta (95% CI)	*p* value
Gender equitable men's scale (GEMS)^b^						
Possible range: 13 to 39	27.6 (26.5, 28.7)	27.0 (26.2, 27.7)	28.0 (27.3, 28.7)	27.9 (27.1, 28.7)	0.45 (−0.87, 1.77)	0.49
	**Weighted % (95% CI)**	**Weighted % (95% CI)**	**Weighted % (95% CI)**	**Weighted % (95% CI)**	**AOR (95% CI)**	***p*** **value**
Sexual behaviour						
Multiple partners in past 12 months	2.8 (1.3, 5.7)	4.6 (2.6, 7.9)	5.4 (3.0, 9.3)	2.6 (1.1, 6.1)	0.29 (0.11, 0.77)	0.01
Condom use at last sex^c^	43.4 (36.6, 50.5)	39.2 (32.0, 46.9)	43.2 (37.6, 48.9)	43.5 (35.3, 52.0)	1.20 (0.70, 2.07)	0.50
Intimate partner violence (IPV)						
Victim of IPV in past 12 months	7.3 (4.3, 12.4)	11.5 (7.7, 16.9)	8.6 (5.9, 12.4)	7.5 (6.2, 9.1)	0.53 (0.24, 1.16)	0.11
Perpetrated IPV in past 12 months	1.4 (0.4, 4.1)	2.8 (1.2, 6.3)	1.3 (0.6, 3.2)	2.9 (1.5, 5.5)	1.06 (0.29, 3.85)	0.93
Substance use						
Hazardous, harmful drinking	0.8 (0.3, 2.8)	1.9 (0.6, 6.5)	1.0 (0.3, 3.7)	3.2 (1.6, 6.2)	1.35 (0.11, 16.87)	0.81

All estimates controlled for age, education, marital status, and received any income in the last three months. AOR, adjusted odds ratio.

a Effect estimates represent the difference between intervention and control at endline, net of the difference at baseline.

b Higher scores represent more equitable views towards gender norms. n = 1199 (1 missing).

c Among those reporting ever having had sex (vaginal or anal), n = 1086.

When examining secondary endpoints for the trial, we did not observe significant differences among men in intervention *versus* control communities over time regarding multiple sex partners in the past 12 months, condom use at last sex, perpetration or experience of intimate partner violence or hazardous drinking (Table 2). However, it is important to note that all secondary endpoints were lower (except condom use, which was higher) in both intervention and control communities. For women, we did observe a significantly lower number of sex partners in the past 12 months in the intervention as compared to control communities, but did not observe significant differences with regard to condom use at last sex, perpetration of IPV or hazardous drinking (Table 3). Women in intervention communities also reported less IPV victimization, although this was not statistically significant (AOR 0.53 95% CI 0.24 to 1.16) (Table 3).

In ancillary analyses we examined the association between outcomes and reported exposure to the intervention activities, or dose received, among those in intervention communities at endline. Overall men were more highly exposed to the intervention compared to women (Tables 4 and 5). The vast majority of men (66.3%) at endline reported medium or high exposure to the intervention (Table 4) compared to less than half of women (41.7%) (Table 5). Men exposed to higher doses of the intervention had higher GEMS scores: 33.7 (low), 35.2 (medium) and 38.1 (high), suggesting a trend towards more equitable gender norms as intervention dose increased. Men exposed to higher doses of the intervention also reported being more likely to have multiple partners, harmful drinking and use condoms than those with lower doses in intervention communities. There was no association of intervention dose and IPV perpetration or victimization among men. Among women, intervention exposure was associated with having multiple partners and greater use of condoms. We did not observe an association between intervention dose and IPV victimization or perpetration, nor with harmful drinking.

**Table 4:** Gender norms and secondary outcomes by level of exposure among men in intervention communities at endline (n = 288)

	Low dose	Medium dose	High dose	
N (%) of sample in category	97 (33.7%)	116 (40.3%)	75 (26.0%)	
	**Mean (95% CI)**			**Adjusted** a ***p*** **value**
Gender equitable men's scale (GEMS)^b^				
Mean (95% CI) Possible range: 17 to 51	33.7 (32.6, 34.9)	35.2 (33.1, 37.2)	38.1 (35.9, 40.3)	0.03
	**Percent (95% CI)**			
Sexual behaviour				
Multiple partners in past 12 months	26.0 (12.3, 39.8)	34.2 (16.3, 52.1)	39.3 (24.7, 54.0)	0.007
Condom use at last sex^c^	34.6 (18.7, 50.5)	70.4 (62.2, 78.6)	60.2 (45.4, 74.9)	0.02
Intimate partner violence (IPV)				
Victim of IPV in past 12 months	3.3 (0.0, 7.1)	8.1 (1.8, 14.3)	3.7 (0.0, 7.6)	0.98
Perpetrated IPV in past 12 months	10.5 (3.9, 17.2)	10.9 (2.8, 19.1)	15.3 (1.5, 29.1)	0.58
Substance use				
Hazardous, harmful drinking	14.1 (4.9, 23.5)	24.9 (16.4, 33.3)	21.9 (10.6, 33.3)	0.0005

a Controlling for age, education, marital status and received any income in the last three months.

b Higher scores represent more equitable views towards gender norms.

c Among men who reported ever having vaginal sex, n = 250.

**Table 5:** Gender norms and secondary outcomes by level of exposure among women in intervention communities at endline (n = 302)

	Low dose	Medium dose	High dose	
N (%) of sample in category	176 (58.3%)	93 (30.8%)	33 (10.9%)	
	**Mean (95% CI)**			**Adjusted** a ***p*** **value**
Gender equitable men's scale (GEMS)^b^				
Mean (95% CI) Possible range: 13 to 39	27.9 (26.6, 29.1)	28.2 (27.0, 29.4)	29.3 (27.7, 30.8)	0.87
	**Percent (95% CI)**			
Sexual behaviour				
Multiple partners in past 12 months	1.3 (0.0, 3.0)	3.7 (0.2, 7.1)	9.9 (0.2, 19.5)	<0.0001
Condom use at last sex^c^	31.8 (24.8, 38.8)	58.6 (45.3, 71.8)	45.7 (14.7, 76.8)	0.02
Intimate partner violence (IPV)				
Victim of IPV in past 12 months	7.2 (4.9, 9.6)	10.0 (2.9, 17.0)	0.4 (0.0, 1.4)	0.64
Perpetrated IPV in past 12 months	2.9 (0.0, 6.1)	2.3 (0.0, 4.9)	5.0 (0.0, 13.0)	0.43
Substance use				
Hazardous, harmful drinking	2.6 (0.0, 5.7)	4.6 (0.0, 9.7)	2.5 (0.0, 8.4)	0.66

a Controlling for age, education, marital status and received any income in the last three months.

b Higher scores represent more equitable views towards gender norms.

c Among women who reported ever having vaginal sex, n = 283.

## 4 Discussion

We conducted a community randomized trial to examine the impact of a theory‐based community mobilization intervention to modify harmful gender norms that place young people at risk of HIV in a high HIV prevalence area. This intervention was successfully implemented across the 2‐year period, as evidenced by monitoring data as well as self‐reported exposure on the endline survey, and contamination was negligible. As hypothesized, in ITT analyses we observed that there was a significant effect of the intervention on our primary outcome, more equitable gender norms, among men, though not among women. The secondary analysis noting that men reporting higher levels of programme exposure at endline also had more equitable gender norms enhances confidence that engagement in the programme led to these changes, though causality cannot be inferred.

In contrast to men, there was no intervention effect on gender norms among women. The One Man Can Intervention was originally intended to be used primarily with men and the primary aim of the study had a key focus on addressing men's gender norms with the aim of reducing HIV risk. However, most of the content and types of activities were highly relevant to women, and the study team and programme implementers adapted the intervention and manuals for use with women. Nevertheless, men had much higher exposure to the intervention compared to women and it may be that types of activities and/or content remained better suited to men. It is also possible that GEMS did not measure the components of the intervention that were most salient to women.

While changing the gender norms environment was the primary goal of our intervention, another important goal was—primarily through changing this normative environment—to reduce key HIV risk behaviours. Research has convincingly demonstrated that men who endorse more harmful gender norms—for example, beliefs that men control decisions around sex, reproductive health and household decisions and are entitled to use violence against women—are more likely to perpetrate IPV, abuse alcohol and have multiple partners, and are less likely to use condoms 10, 12, 52, 53, 54, 55, 56, 57, 58. However, while we observed changes in normative beliefs among men, we did not observe significant differences in gender‐based violence or HIV risk behaviour among men.

ITT results for intervention effects on IPV—namely that the intervention was associated with lower, albeit non‐significant, IPV victimization among women, while there was no effect for men—are similar to those found in other recent mobilization trials in Uganda aiming specifically to reduce gender‐based violence. In the SASA community mobilization intervention trial, which was focused on reducing IPV, the investigators found a significant reduction in the acceptability of IPV among men and women and lower levels of women reporting physical and sexual violence victimization, however the latter results were not statistically significant 14, 59. Ancillary analysis in the SASA trial, to assess how the intervention reduced IPV found that community level norms around the acceptability of IPV explained 70% of the intervention's impact on women's experience of IPV and 95% of the impact on men's perpetration 59. In the SHARE trial in Uganda that focused on IPV and HIV risk reduction, a statistically significant reduction in women's victimization of IPV was observed but a reduction in male perpetration of violence was not 60. The SASA and SHARE interventions focused primarily on IPV reduction while our intervention was more broadly focused on gender norms in the context of HIV prevention, although activity and workshop content included modules on IPV. However, other trials in South Africa aimed at both reductions in IPV and HIV, such as the IMAGE and Stepping Stones trials, did demonstrate reductions in IPV perpetration among men (Stepping Stones) and victimization among women (IMAGE) 6, 61. These interventions enrolled cohorts of individuals to participate in multiple sessions over time (e.g. Stepping Stones involves 12 sessions over time) which also may account for changes.

Also of note, in ancillary analyses among the intervention group at endline, men reporting more intervention exposure also reported more sexual partners and more hazardous drinking than those with less programme exposure, although they also reported more condom use. Similarly, women with greater intervention exposure also reported more multiple partners but also more condom use than women with less intervention exposure. It is unlikely, given the programme content, that the intervention resulted in increased partner number or problem drinking, rather these findings may indicate that the programme attracted young people who were more social: more likely to be sexually active and to drink regularly. In particular, some of the outreach activities targeted individuals in drinking venues. It is also possible that since the programme content dealt with HIV prevention and norms within relationships that individuals who had partners and were sexually active were more likely to participate.

There are a number of reasons why this intervention may not have had an impact HIV risk behaviours and gender‐based violence. First, the programme may not have sufficiently focused on promoting or supporting actionable steps related to changing the behaviours of interest. While workshops and activities were quite detailed in assisting participants to think through the nuances of gender and how norms are enacted and can be changed or challenged, activities did not all encompass concrete action steps to change behaviour. Second, it is possible that the dose of the intervention achieved by individuals within the community was not enough to lead to behaviour change or that enough intervention time had not elapsed to allow for changes in norms to translate into changes in behaviour.

Third, certain theoretical domains of community mobilization on which the programme was based were better achieved than others. The model of community mobilization focuses on first raising awareness through building community consciousness, in this case around the intersection of inequitable gender norms and HIV. We did this through more formal workshop activities, through informal one‐on‐one interaction between the community mobilizers and community action teams and their communities, and through programme activities. This aspect of the model was well implemented per our monitoring data as well as evidence of widespread programme exposure in the endline survey. However, determining how to realize other aspects of the model may require further development. For example, two other integral components of mobilization involve engaging with community leaders and capitalizing on organizations and networks to ensure message dissemination throughout the process 16, 28. Engaging leadership and formal organizations proved challenging in the present intervention, with a largely youthful community‐based mobilization team in an area where formal leadership often comprises tribal elders and formal organizations align closely with leadership structures. Future interventions should explore the role of informal networks, and seek to modify engagement with leaders into concrete commitments to achieving measurable programme targets.

### 4.1 Limitations

This study has a number of limitations. First, while repeated cross‐sectional studies are good for measuring changes at a community level over time, it is possible that differences in the population sampled at each time point may account for differences (or lack of differences) observed. While using repeated cross‐sections for cluster randomized trials provides the opportunity to measure community level change, it does not allow for examining change in individuals exposed to the intervention overtime that a cohort design provides. Second, GEMS scores among men were non‐equivalent across intervention and control communities at baseline. While our intent‐to‐treat analyses accounted for differences in outcomes by arm at baseline, such differences may still be important information to consider when interpreting trial results. Third, trial outcomes were based on self‐reported survey data, which may be prone to social desirability. Attitudes as study outcomes for interventions aiming to change negative norms may be particularly prone to social desirability bias and also may not be reflective of actual behaviours. While low GEM scores have been associated with risk behaviour in multiple settings and have been shown to change in relation to gender transformative interventions, it is possible that there are better, more proximate measures to capture gender norms or that proceed reductions in GBV and other risk behaviours. Fourth, our ancillary exposure analyses contribute to our understanding of how direct personal exposure to the intervention may influence outcomes; however, causality cannot be inferred due to the cross‐sectional nature of the exposure data. Fifth, our study was not powered to detect changes in behaviour. In addition, while our study was powered to detect at 10% change in the GEM score, it is unclear what level of change in GEMS would result in behaviour change and thus what a clinically meaningful change would be. Finally, reaching men and women consistently with the intervention over the full intervention period was challenging. Migration for work purposes is common in this area with as many as 60% of adult men and 30% of women leaving home to find work in any given year 62. This may have implications for consistent exposure to the intervention and could deter community change.

## 5 Conclusion

Overall we found that this intervention to change harmful gender norms was successful in doing so among men, and those men who had greater exposure to the programme held more equitable gender norms than those with lower exposure. Despite improvements in men's gender norms, behavioural outcomes among men and women remained largely unchanged between intervention and control communities, which could signal that either our approach or our measure of gender norms did not sufficiently address aspects of gender that lead to changed behaviours or that there was insufficient time for attitudes to translate into behavioural change. In future iterations of gender‐transformative interventions, we recommend that programme content be altered to address more explicit action‐oriented steps related to partner reduction, IPV perpetration prevention, and harmful drinking and a longer intervention timeline (greater than two years). Nevertheless, CM has demonstrated potential to create environments that are more supportive of preventing gender‐based violence and other HIV‐risk factors.

## Competing interests

The authors have no conflicts of interest to declare.

## Authors contributions

AP, SL, KK, ST and CM developed the study design, raised funds for the trial and oversaw implementation of the study, interpreted study findings and wrote up the findings.

AG and CS developed the analysis plan and analysed the data and helped write the manuscript

DP, DR, AG and SM contributed to intervention development and oversaw implementation of the intervention and contributed to the main finding interpretations and edited the manuscript.

RT, FXG and AS oversaw implementation in the field, conducted study feedback and contributed to interpretation of finding and edited the manuscript.

## Supporting information


**Table S1.** Summary implementation data for two‐year Community Mobilization intervention (May 2012 to April 2014)
**Table S2.** OMC mobilization activities mapped onto domains of community mobilization

**Appendix S1.** Among men and women, primary outcome adjusted effect estimates using GLMM.
