# Supplementary material for: Community mobilization to modify harmful gender norms and reduce HIV risk: results from a community cluster randomized trial in South Africa
Source: J Int AIDS Soc. 2018 Jul 4;21(7):e25134. doi: 10.1002/jia2.25134 (PMC6058206; doi:10.1002/jia2.25134)
Supplement: Supplementary file 1 — Table S1. Summary implementation data for two‐year Community Mobilization intervention (May 2012 to April 2014) Table S2. OMC mobilization activities mapped onto domains of community mobilization [file JIA2-21-e25134-s001.docx]

Additional Files.

Table 1s. Summary implementation data for two-year Community Mobilization intervention (May 2012 to April 2014)

|  | **Workshops conducted** | |  | **Activities Conducted** | |  | **# Leadership Engagement Meetings** | |  | **# Community Action Team (CAT) Meetings** | |  | **Percentage of male pop. age 18-35 reached with at least 1 workshop (cumulative)** | |
| --- | --- | --- | --- | --- | --- | --- | --- | --- | --- | --- | --- | --- | --- | --- |
|  | **Yr 1** | **Yr 2** |  | **Yr 1** | **Yr 2** |  | **Yr 1** | **Yr 2** |  | **Yr 1** | **Yr 2** |  | **Yr 1** | **Yr 2** |
| ***Larger Villages*** |  |  |  |  |  |  |  |  |  |  |  |  |  |  |
| Community 1 | 15 | 27 |  | 277 | 367 |  | 8 | 6 |  | 42 | 44 |  | 15% | 34% |
| Community 2 | 13 | 26 |  | 228 | 300 |  | 8 | 5 |  | 41 | 44 |  | 17% | 34% |
| Community 3 | 16 | 23 |  | 304 | 602 |  | 12 | 8 |  | 50 | 77 |  | 11% | 27% |
| Community 4 | 13 | 22 |  | 179 | 257 |  | 2 | 5 |  | 42 | 43 |  | 11% | 27% |
| Community 5 | 15 | 29 |  | 183 | 314 |  | 7 | 7 |  | 41 | 49 |  | 11% | 32% |
| Community 6 | 11 | 27 |  | 187 | 285 |  | 7 | 9 |  | 40 | 46 |  | 16% | 43% |
| ***Smaller Villages*** |  |  |  |  |  |  |  |  |  |  |  |  |  |  |
| Community 7 | 8 | 11 |  | 165 | 314 |  | 4 | 4 |  | 36 | 46 |  | 35% | 51% |
| Community 8 | 11 | 12 |  | 170 | 236 |  | 9 | 5 |  | 41 | 47 |  | 20% | 34% |
| Community 9 | 12 | 11 |  | 204 | 328 |  | 10 | 10 |  | 42 | 48 |  | 17% | 45% |
| Community 10 | 9 | 13 |  | 167 | 294 |  | 8 | 8 |  | 41 | 47 |  | 21% | 39% |
| Community 11 | 12 | 13 |  | 210 | 259 |  | 7 | 8 |  | 43 | 47 |  | 25% | 44% |
| **Total per year** | **135** | **214** |  | **2,274** | **3,556** |  | **82** | **75** |  | **459** | **538** |  | **18.1%** | **37.3%** |
| **TOTAL** | **349** | |  | **5,830** | |  | **157** | |  | **997** | |  | **37.3%** | |

Table 2s. **Additional description of the OMC intervention**

The OMC intervention was designed to facilitate engaged dialogue around gender norms that place both men and women at risk of HIV and to increase men’s engagement with protective behaviors through community mobilization (CM) activities. The CM activities, in addition to encouraging community dialogue, were created to bring together organizations and networks to disseminate intervention messages, to engage leadership, foster community cohesion to support gender norm change and HIV prevention, and to facilitate community action with community action teams (CATs). Table 2S includes a list of OMC activities mapped onto the domains of community mobilization.

**Table 2s.** OMC mobilization activities mapped onto domains of community mobilization.

|  | **Shared concerns** | **Critical consciousness** | **Engage leadership** | **Orgs / networks** | **Collective action** | **Social cohesion** |
| --- | --- | --- | --- | --- | --- | --- |
| 2-day OMC workshops | X | X |  |  |  | X |
| OMC mini workshops | X | X | X |  |  |  |
| Shebeen workshops | X | X |  |  |  |  |
| Red card campaigns |  | X |  |  | X |  |
| Picture charts for discussion | X | X |  |  |  |  |
| Condom distribution | X | X |  |  |  |  |
| Door to door campaign | X | X |  |  |  |  |
| Street soccer / Soccer tournaments | X | X | X | X |  | X |
| Painting community murals |  | X |  |  | X | X |
| Ambush theatre | X | X |  |  |  |  |
| Digital stories workshops | X | X |  |  |  | X |
| OMC Action Sheets | X | X |  |  | X |  |
| Establish / train CATs |  |  | X | X |  | X |
| Meetings with community leaders |  |  | X | X |  |  |
| Photo voice | X | X | X |  | X | X |

The intervention team carried out activities, focusing first on smaller group activities and workshops that focused on raising critical consciousness and generating a shared concern, and later moving onto larger community forums to elicit action steps and broader discussions. Early in the intervention, individuals who participated in activities were identified as potential Community Action Team (CAT) members and trained by the team of mobilizers in each village. Mobilizers worked closely with CAT teams as they implemented activities. CATs met weekly to plan activities and workshops, to discuss progress and challenges in engaging community members, and to learn new content / undergo additional training and exercises with the mobilization team.
