# Supplementary material for: Community mobilization to modify harmful gender norms and reduce HIV risk: results from a community cluster randomized trial in South Africa
Source: J Int AIDS Soc. 2018 Jul 4;21(7):e25134. doi: 10.1002/jia2.25134 (PMC6058206; doi:10.1002/jia2.25134)
Supplement: Supplementary file 2 — Appendix S1. Among men and women, primary outcome adjusted effect estimates using GLMM. [file JIA2-21-e25134-s002.docx]

**Appendix. Among men and women, primary outcome adjusted effect estimates using GLMM**

|  | **Adjusted effect estimate*** | |
| --- | --- | --- |
| **Characteristics** |  |  |
|  | *Beta*  *(95% CI)* | *P value* |
| **MEN: Gender Equitable Men’s Scale (GEMS)** | 2.75  (0.79 4.72) | 0.01 |
| **WOMEN: Gender Equitable Men’s Scale (GEMS)** | 0.33  (-1.25, 1.92) | 0.68 |

*All estimates controlled for age, education, marital status, and received any income in the last three months.
